# Barriers to and Facilitators for Finding and Keeping Competitive Employment: A Focus Group Study on Autistic Adults With and Without Paid Employment

**DOI:** 10.1007/s10926-024-10181-3

**Published:** 2024-04-03

**Authors:** Evelien P. M. Brouwers, Michel Bergijk, Jaap van Weeghel, Sarah Detaille, Hanneke Kerkhof, Jeroen Dewinter

**Affiliations:** 1 Tranzo Scientific Center for Care and Wellbeing, Tilburg School for Social and Behavioral Sciences, Tilburg University, Tilburg, The Netherlands; 2 Research Department Human Capital Innovations, HAN University of Applied Sciences, Nijmegen, The Netherlands; 3 HumanCapitalCare b.v., Son, The Netherlands; 4 GGzE Centre for Child and Adolescent Psychiatry, Eindhoven, The Netherlands

**Keywords:** Autism, Employment, Barriers and facilitators, Focus group, Workplace inclusion, Self-insight

## Abstract

**Purpose:**

The aim of the study was to gain more insight into barriers to and facilitators for finding and keeping competitive employment for autistic adults. Research questions were: (1) What barriers and facilitators do autistic adults report in finding and keeping competitive employment?; and (2) What are differences and similarities between autistic adults with and without paid employment regarding barriers and facilitators for sustainable employment?

**Methods:**

Eight focus groups were conducted (*N* = 64 autistic adults). Four groups included only participants *without paid employment *(*N* = 24), and four groups consisted exclusively of participants *with* current paid employment (including part-time, *N* = 40). All discussions were audiotaped and transcribed verbatim to enable inductive thematic content analysis. Data were analyzed using ATLAS.ti 9.

**Results:**

Ten themes and thirty-four subthemes were found. Many were interconnected. Themes facilitating sustainable employment included a positive workplace atmosphere, a supportive supervisor, being able to do work that aligns with interests and talents, favorable physical working conditions, coaching, higher self-insight, higher self-esteem, and proactivity. Most themes and subthemes emerged from both groups. Differences between the groups were that those *with* paid employment seemed to have experienced more friendly workplaces and supervisors, had received better coaching in finding and keeping employment, had higher self-insight and higher self-esteem, were more assertive and proactive.

**Conclusions:**

As many (sub-)themes were interrelated, the results suggest that to improve work participation, particularly two key areas are promising: (1) to realize more friendly, well-being oriented and inclusive workplaces, and (2) to increase autistic adults’ self-insight into personal needs for positive wellbeing and self-knowledge regarding talents, wishes and well-being boundaries.

**Supplementary Information:**

The online version contains supplementary material available at 10.1007/s10926-024-10181-3.

## Introduction

Despite efforts to improve employment outcomes for autistic adults, internationally their employment rates remain low. For instance, according to the Netherlands Autism Register, only 48% of autistic adults reported to have paid employment in 2021 [1] compared to 72% of the general population [2]. In the UK, recent numbers from the Office for National Statistics showed employment rates of autistic adults to be only 29% in 2021 [3]. Also, sustainability of employment is often a challenge, as many of those who have competitive employment have discontinuities in their careers [4, 5].

These low employment rates are problematic, given the benefits of (decent) employment for mental health and well-being [6, 7] and the high costs of lost productivity [8]. A scoping review showed that interventions so far have mainly been individual and impairment- focused, trying to ‘fix’ the autistic adult [9], and overlooking the role of the work environment, as well as strengths, talents, and preferences. Hence, a broader and more holistic research scope is needed. In the present study, the aim was to study barriers and facilitators for sustainable employment in two separate groups of autistic adults: those with and without paid (competitive) employment. Studying these two groups separately (yet under the same legislation and in the same country and culture) may yield a clearer picture of important barriers and facilitators then when autistic adults are studied as one group. Specifically, the research questions of this focus group study were: (1) What barriers and facilitators do autistic adults report in finding and keeping competitive employment?; and (2) What are differences and similarities between autistic adults with and without paid employment regarding the barriers and facilitators they report for sustainable employment?

## Method

We conducted eight focus groups (*N* = 64) in November and December 2019. Four focus groups exclusively entailed participants who **had paid (competitive) employment** at the time of the focus group (*N* = 40), and the other four focus groups entailed participants **who did not have paid employment** at that moment (*N* = 24). Group sizes ranged from 13 to 4, each participant taking part only once. The discussions lasted two hours, of which the first 30–60 min were used for a different study, on the meaning of paid employment for wellbeing [6]. We reimbursed travel costs and participants received a gift certificate of 10 euros for taking part in the study. We followed the Consolidated criteria for reporting qualitative research (CORECQ) guideline. The CORECQ checklist [10] can be found in Supplementary.

### Participants

Participants were eligible if they reported to have received a formal Autism Spectrum Disorder diagnosis from a psychiatrist or psychologist. We used convenience sampling, recruiting participants through work re-integration specialists from a large mental health care institute, occupational physicians, members of the Dutch autism association (NVA), Twitter, LinkedIn and during the annual Dutch ‘Autminds’ conference. After receiving more information about the study, 27 people declined participation for various reasons: unavailable at the times and dates of the focus groups (*N* = 10), traveling would cost too much energy or was too far (*N* = 6), no interest in the study (*N* = 9), did not show up (*N* = 2). All 64 participants were Dutch Caucasians, of whom 36 were female. Most were highly educated, with 39 having an (applied) university degree. Twenty-eight participants were single. The mean age was 47 (SD 10,98). Participants with paid employment worked in a variety of different professions, e.g. as a lawyer, systems engineer, cleaner, schoolteacher, and assistant pharmacist.

### Focus Groups

We held the meetings at three different locations in The Netherlands: a university, a mental health knowledge institute, and a mental health care organization. All groups were guided by two researchers. MB, a male junior researcher (MSc) was present at all eight meetings and had the role of observer. EB, and JD (both PhD and experienced in qualitative research) each lead four focus group discussions. One participant brought a personal coach to a focus group, who did not participate in the conversation. During and after the meetings, EB and MB made field notes. Each meeting started with an introductory round during which the researchers introduced themselves as well, informing participants that JD was a psychologist specialized in autism, and MB was autistic. Next, we explained the goals of the study, and answered remaining questions. The topic list can be found in Table 1. We did not pilot test the topic list and did not ask participants for member checks or feedback on the results. After eight focus groups, we did not gather any new information related to our research questions, and data saturation was reached.

**Table 1:** Topic list

1. How easy or difficult is it for you to **find** paid employment?
2. What makes it *difficult* for you to **find** paid employment, and why?
3. What makes it *easy* for you to **find** paid employment, and why?
4. How do you deal with barriers in **finding** paid employment?
5. How do you deal with facilitators in **finding** paid employment? 6. What is the effect of these barriers and facilitators on how you feel?
7. What is the effect of these barriers and facilitators on your sustainable employability?
8. How easy or difficult is it for you to **keep** paid employment?
9. What makes it difficult for you to **keep** paid employment, and why?
10. What makes it easy for you to **keep** paid employment, and why?
11. How do you deal with barriers in **keeping** paid employment?
12. How do you deal with facilitators in **keeping** paid employment?
13. What is the effect of these barriers and facilitators on how you feel?
14. What is the effect of these barriers and facilitators on your sustainable employability?

### Coding, Data Analysis and Interpretation of the Data

We audiotaped all meetings and transcribed them verbatim. Before the analyses were performed, EB anonymized all transcripts. We conducted deductive and inductive thematic content analysis at a semantic level, following an interpretivist phenomenological paradigm [11, 12]. The first research question (on barriers and facilitators for finding and keeping paid employment) was used as a framework, with pre-defined categories of ‘barriers’ and ‘facilitators’. Subsequently, themes and subthemes within these predefined categories were created by the method of constant comparison, in which different codes were compared and relationships between codes were explored to detect emerging themes and subthemes [11]. This process was done by EB who clustered the codes and defined emerging themes. To augment reliability, each transcript was read repeatedly, and coded by 2–3 researchers independently (EB, MB, JD, JvW, SD). Codes were created using open, axial, and selective coding [13]. To answer the second research question, (i.e. to identify differences and similarities between the groups) data were analyzed separately, hence resulting in separate code lists and emergent themes for the groups with and without employment. In case comparable themes emerged in both types of focus groups (e.g. ‘workplace atmosphere’), the same theme titles were used to enhance visibility of similarities and differences. For the data analysis, EB used the software program ATLAS.ti version 9.

### Ethical Considerations

Prior to the study, the study proposal was reviewed and approved by the Ethics Review Board of Tilburg University (number EC 2019.68). During recruitment as well as at the start of the focus group meetings, MB, EB, and JD provided written and verbal information about the study and answered questions. During the start of the focus group meetings, we thereafter asked participants to sign an informed consent. Here, none of the participants declined consent or participation.

## Results

Concerning barriers and facilitators for finding and keeping paid employment a total of ten themes and 34 subthemes were found. Most themes were found in both groups, although some differences emerged between those with and without employment. An overview of the themes and differences can be found in Table 2.

**Table 2:** Themes and subthemes, and differences in barriers and facilitators

Themes	Subthemes	Barrier or facilitator	Discussed in groups **with **work	Discussed in groups **without** work
Workplace atmosphere	Being surrounded by friendly and positive people	Facilitator	+	+
	Feeling appreciated at work	Facilitator	+	+
	Feeling/being part of a team	Facilitator	+	+
	Being allowed to be different and authentic in the workplace	Facilitator	+	+
	A lack of knowledge about autism and prejudice in the workplace	Barrier	+	+
	Unwillingness and inflexibility from people in the workplace (e.g. HR managers, colleagues) to make tailored adjustments that would benefit the autistic worker’s needs	Barrier	+	+
	Social exclusion and discrimination of the autistic worker	Barrier	+	+
	Social turmoil and tension in the work environment	Barrier	+	+
Supervisor skills and attitudes	Supervisor who has a positive/inclusive attitude	Facilitator	+	+
	Supervisor who pays attention to worker’s wellbeing and who protects workers against stress	Facilitator	+	+
	Supervisor who does not listen to what the autistic worker needs	Barrier	+	–
	Unwillingness and inflexibility from supervisors to make personalized exceptions that benefit the autistic worker’s needs	Barrier	+	+
	Under- or overestimation of the autistic worker’s skills and capacities	Barrier	+	+
Interesting versus uninteresting work	Being allowed to do work tasks that align with interests and talents	Facilitator	+	+
	Talents are seen and acknowledged by others in the workplace	Facilitator	+	–
	Worker is allowed NOT to do things that cost negative energy	Facilitator	+	–
Working conditions	Clarity and structure in work tasks and social expectations	Facilitator	+	+
	Autonomy regarding work tasks and speed	Facilitator	+	+
	Workspace free of disturbing stimuli (e.g. noise, light)	Facilitator	+	+
Support in finding or keeping employment (e.g. coaching)	Receiving helpful support in finding or keeping paid employment	Facilitator	+	+
	Having learned useful strategies and tricks from previous coaching e.g. in dealing with problems at work	Facilitator	+	–
	Feeling the need for support in finding or keeping employment but not receiving any	Barrier	–	+
	Receiving inadequate or unhelpful support	Barrier	–	+
Self-insight and self-knowledge	Not understanding why trying so hard did not yield the desired results, and costed so much negative energy, prior to autism diagnosis	Barrier	+	+
	Ignoring boundaries (e.g. work-life balance) and working too hard	Barrier	+	+
	Not knowing own talents or job preferences	Barrier	–	+
Self-esteem and assertiveness	Assertiveness to supervisor regarding one’s needs	Facilitator	+	–
	Low self-esteem	Barrier	–	+
Proactivity versus passivity/fatigue	Proactive attitude and behavior	Facilitator	+	–
	Lack of energy	Barrier	–	+
Communication	Good mutual communication between worker and supervisor about the workers’ needs	Facilitator	+	–
	Implicit social rules and employers’ expectations during job applications	Barrier	+	+
	Autistic worker being too honest	Barrier	+	+
Disability benefits trap	Receiving disability benefits provides income, but fear of losing these benefits hampers re-entry in the job market	Barrier	–	+

### Theme 1: Workplace Atmosphere

Both groups emphasized that *a positive and inclusive work atmosphere* was a crucial facilitator for finding and keeping paid employment. Four facilitating subthemes were found: (1) *Being surrounded by friendly and positive people*; (2) *Feeling appreciated at work*; (3) *Feeling/being a part of the team;* and *(4) Being allowed to be different and authentic in the workplace*. The following quote from a participant who was thriving at his paid job illustrates the importance of these aspects:

“So I was afraid that nobody would want to work with me because I had been diagnosed as autistic [..]. But when I told my close coworkers, they said: ‘We’ve known this for a long time already […] and it is okay. That is why you have created such a fine calculation model’”

*Participant with employment*

In contrast, in both groups, *A negative and excluding work environment* was seen as a major barrier to sustainable employment, although this theme was most elaborately discussed among those with employment, who had more experiences in this area. Four subthemes were found. The first was: *A lack of knowledge about autism, and prejudice in the workplace,* with which many participants had personal experience. The following two quotes illustrate how this subtheme forms a problem:

“Asking attention for autism [in the work environment] is very difficult, because they don’t understand. I get the famous 10 remarks like: ‘O you can’t really tell you are autistic’, ‘you are not very autistic’, ‘I have that as well’, and so on. In fact, those are all downplaying remarks that make it difficult for me to say: ‘This or that really bothers me’. It makes me a bit of a whiner or an attention seeker”.

*Participant without employment*

“I very frequently encounter prejudice. People at work who just have an image of autism based on for instance *Rain Man*, which they saw on television.[ …] I am open about my autism so I get fewer opportunities at work. Many believe that if you’re autistic, you can’t do the work. That is really hindering me”.

*Participant with employment*

The second subtheme related to a negative work atmosphere was *Unwillingness and inflexibility from people in the workplace to make personalized exceptions that benefit the autistic worker’s needs*. Even if participants communicated assertively about their needs, they often found workplace stakeholders (e.g. supervisors, coworkers, human resources personnel) unwilling to adjust their own behavior or existing work routines. This was seen by participants as a missed opportunity to prevent stress and to stay at work sustainably.

“[I try to be assertive …] but reactions are not always positive. […] For instance when I say: ‘this is really hard for me, can we please see if anyone else can do it?’, I have a colleague who [cynically] says: ‘Well hello, you have a normal job and we all do it this way, and so will you”.

*Participant with employment*

“When I said [..] I need more calmness and preferably a workspace of my own, or somewhere in a corner [..] they said: No, we won’t engage in such practices”.

*Participant with employment*

The third subtheme relating to a negative work environment was *Social exclusion and discrimination of the autistic worker,* which many participants had personal experience with, particularly in the group with employment. Especially hiring discrimination was commonly experienced, often after disclosure (e.g. not being hired, or being fired).

“So when I was forced to disclose my autism […] in fact it was from that moment on that they started to try to get rid of me. […] At the consultancy agency I hardly was assigned any projects anymore. I was not allowed to visit clients anymore because I was seen as unreliable”.

*Participant without employment*

“[Shortly after my diagnosis..] I applied for jobs at 20 companies, all ICT companies, […]. And each time when I said: ‘There is something that you should know about me, which is perhaps good for you to know’, I was rejected immediately. Whereas verbally they basically had already hired me”.

*Participant with employment*

The fourth subtheme found relating to a negative workplace atmosphere, was *Social turmoil and tension in the work environment.* In both groups, many believed that their sustainable employability was negatively influenced by social tensions in the team, office politics, social power games, hidden agendas, or upcoming reorganizations.

### Theme 2: Supervisor Skills and Attitudes

In both groups, the crucial role of a good supervisor for sustainable employment was highlighted. Two subthemes were found relating to a facilitating supervisor: (1) *having a positive/inclusive attitude*; and (2) *paying attention to workers’ wellbeing and protecting workers against stress*. In contrast, both groups indicated that supervisors can also be a major barrier to sustainable employment. Here, three subthemes were found. The first was *Supervisors who do not listen to what the autistic worker needs*. This referred for instance to supervisors who ‘already know what’s best’ for the worker without discussing this with the worker him/herself.

“For a year and a half I had been telling my supervisor that this work was not challenging enough for me [..] But my supervisor did not really listen to what I had to say. I told him it was too stressfull […] They were satisfied with my work performance but did not see that I was breaking down [..] Eventually I fell ill because they just totally did not listen to me”.

*Participant with employment*

“The biggest problem always returns: supervisors don’t ask employees, they ‘already know’ what they [employees] need. And that is where things go wrong”

*Participant with employment*

The second subtheme was *Unwillingness and inflexibility to make personalized exceptions that benefit the autistic worker’s needs:*

“When I had such high work stress that I became sick [..] I told my (former) employer: one of the things I need for now is that I get a written overview of the order in which I need to do my work tasks. The reaction to that was: ‘How ridiculous, […] of course that is not possible’. At my current job […] I asked my supervisor: ‘Can you please send me an email of what you want me to do? [..] Five minutes later I had her email”.

*Participant with employment*

The third subtheme related to unhelpful supervisor behavior was ‘*under- or overestimation of the autistic worker’s skills and capacities*’. This subtheme was very common in both groups and viewed by participants as negatively affecting their sustainable employment. The quotes implied that the supervisor did not see their true capacities and skills level, and that this was not a topic of conversation between supervisor and employee.

“As soon as you start to function less well because of stress, they [supervisors] take away work tasks without considering if they are tasks that give you positive energy. So you get a boring job, instead of that fun project you are so motivated to work for. […] They mix up overstimulation and positive challenge”.

*Participant with employent*

“People with Asperger’s […] always get jobs below their education level. The idea behind that is by working at a lower level, they won’t get overstimulated and will be able to work sustainably. [..] But I know from experience that if you work below your education level, you get frustrated, which eventually will lead to drop out. […] “

*Participant with employment*

### Theme 3: Interesting Versus Uninteresting Work

Doing work that aligns with interests and talents, which is enjoyable, and which gives positive energy was seen as an important facilitator for sustainable employment in both groups.

“I love my job, because technology is my passion, and that is what I am doing. And I have fun coworkers, who also work in engineering, and we make jokes about technology, and during the breaks we talk about technology, it’s simply wonderful”.

*Participant with employment*

Only in the groups with paid employment, much was said about two additional subthemes. First, employed participants often said they really enjoyed the content of their work or their work tasks, and that *their talents were seen and acknowledged by others in the workplace*. For instance, they were complimented by their supervisor, or approached by recruiters from competing companies. Second, they *were allowed NOT to do things that cost them negative energy*:

“The only reason why I still work there is that I don’t do tasks that don’t suit me. Then I discuss with my supervisor who else could do it, and he will know someone who will really enjoy doing that particular task. […]”.

*Participant with employment*

“[Together with a close coworker I own a company, and things are going really well at work because] my coworker deals with the more socio-emotional and human resources aspects of our work. That means I can fully focus on the content [..] and use my strengths optimally”.

*Participant with employment*

### Theme 4: Working Conditions

In both types of groups, three subthemes relating to working conditions emerged. The most discussed one was the need for *Structure and clarity in work tasks, procedures, and social expectations*. A lack of structure and clarity was commonly discussed as disturbing and energy draining, especially non-adherence to social agreements or promises by coworkers or supervisors.

“I don’t always feel good at work because of the high unpredictability and chaos that often destroy my planning. It is a huge energy drain. A few years ago, when I had an organized administrative job […] I would put on my running clothes after work and would go for a 6 miles’ run. [..] But now, after one of those days, I don’t have the energy, and I regret that”.

*Participant with employment*

The second subtheme was *Autonomy regarding work tasks and speed,* and the third was the preference for a *Workspace free of disturbing stimuli* (e.g. noise, light). Some mentioned that they found it difficult to work in open-plan offices, and others talked about their preference for a permanent (rather than flex-) workspace.

### Theme 5: Support in Finding and Keeping Paid Employment

In both groups, the importance of having a (job) coach was stressed as a highly important facilitating factor for finding and keeping employment. *Helpful support* was for described as having someone with whom to discuss difficult social situations, who can provide help in creating clearity and structure, and setting boundaries to create a healthy work-life balance. A subtheme found in those *with employment* only, was that they *had learned useful strategies and tricks from previous coaching in dealing with problems at work:*

“My coach explained to me what overstimulation is and what can be done against it. I still use those tips. For example, during my lunch break I prefer to eat alone. I have explained that to my coworkers, everybody knows [..]. It is my moment of rest”.

*Participant with employment*

There were two important subthemes related to the theme *Support*, that only emerged in the groups *without employment*. First, participants in these groups ofen discussed that *they felt a need for support, whilst not receiving any*. For instance, they expressed a need for support in how to apply for a job and how to behave during the job interview, but also how to start a new job, how to plan and prioritise work tasks, take next steps, and how to make work related choices. A second subtheme in the groups *without* employment was that they had often received *inadequate or unhelpful support*. Participants often felt their re-integration professionals did not (want to) understand them, lacked knowledge about autism or were pushy, which hampered their (re-)employment opportunities.

“There is a re-integration coach at my former employer who I can consult, now that I have been declared work disabled. [I don’t do this because] he said to me: you don’t look like you are ill at all”.

*Participant without employment*

### Theme 6: Self-Insight and Self-Knowledge

Participants in both groups often discussed they tended to have little self-insight and self-knowledge, which was a barrier to sustainable employment. Prior to receiving their autism diagnosis, many had known for a long time that there was something ‘different’ about them. When they recieved the autism diagnosis (often after an episode of burnout) this clarified many things for them, also from the past. Such as why some things had been such a struggle, or why they had been treated differently (e.g. bullied) in previous jobs or in school. Not knowing why they were different from most others had negatively affected their wellbeing, health, and occupational success, and this was one of the most elaborately discussed themes found in this study, because it often had a strong negative effect on well-being and employment.

“I tried my absolute best but it wasn’t enough. Why could I not do [hold a job], because [..] intellectually I should be able to handle it. I also failed at jobs below my [education and skills] level. If you don’t know why you do not succeed, that is really hard”.

*Participant without employment*

“I had had two previous burnout episodes of which the cause was unclear. When I got the third, I received the diagnosis, which clarified much of why I had a breakdown again”

*Participant without employment*

*“There have been years where I was continuously swearing and cursing at work. Where I thought: apparently I am not no good, or different, or abject and something is going horribly wrong, but I never got an answer to what was going on. Because I am good at my work, they tolerated it, but it has considerably damaged my mental wellbeing and happiness”*

*Participant with employment*

A difference between the groups was that those without employment seemed more damaged by these past experiences, and often described them in terms related to suffering, using words like “hell” and “being broken”. For them, receiving their diagnosis was sometimes described as a long awaited acknowledgement for their suffering and a reason to give up on further efforts to find or keep paid employment. The moment of receiving the diagnosis was also described as a turning point for participants’ careers in terms of job loss due to discrimination, or alternatively sometimes to improved relationships at work.

A second important subtheme related to the theme *Self insight and Self-knowledge* was: *Ignoring boundaries and working too hard.* Especially in the group without employment, participants discussed that they did not know their own boundaries (e.g. work-life balance) and that it had left them depleted at some point.

“I had ignored my boundaries for years [at work], and when several things [in my private life] happened I got stuck […] and now I am chronically fatigued”.

*Participant without employment*

“I worked as a software engineer, on average 50-100 hours a week, for 15 years. Worked in Iran, everywhere across Europe [..]. I did however drop all my social contacts and sports [..] So I came home on Fridays, went to bed and woke up on Mondays. I managed to continue to do this for 15 years and then I fell over”.

*Participant with employment*

A third subtheme related to self-knowledge was that those without paid employment often discussed that they did not know *what their talents were or what kind of work they would like to do.* This made it more difficult to find employment. In contrast, those with paid employment seemed to have somewhat more self-knowledge about what caused them stress, what helped them to function and feel well and what their talents were.

### Theme 7: Self-Esteem and Assertiveness

Much was said relating to self-esteem and assertiveness, and important differences between the groups were found. In the groups without employment, many indicated they had been bullied at school or had been told by others they were ‘stupid’ during their youth, which had resulted in low self-esteem. They often felt distrust towards supervisors or coworkers and felt they had been taken advantage of in past work experiences. Negative experiences had scarred their self-esteem which hindered them in finding and keeping employment.

“Actually, in terms of intelligence, I could well have paid employment. It is juist my anxiety and insecurity, the idea of not being able to live up to expectations. That is what limits me mostly”.

*Participant without employment*

In contrast, participants in the groups with employment said things that showed that they stood their ground and were assertive, despite their insecurities:

“After my diagnosis […] I said to my supervisor: ‘I would like you to come sit in my class and attend a lesson, and I want you to give me feedback on two things. You will tell me what I need to continue doing, and what I absolutely need to change”

*Participant with employment*

Especially if they were in an environment that was open to their assertiveness, this could yield positive results. The following quote from an autistic participant who reported to be doing well at work illustrates this:

“When I got the diagnosis […] my employer suggested that I should get coaching, so I could start to do certain things differently, which I thought was fair. I told my employer that I certainly was open to coaching, but that I thought it had to come from both sides. My supervisor also found it difficult to communicate with *me*, so I said: ‘then I would like it if he also gets coaching’. So the same coach had sessions with me *and* my supervisor, and I actually appreciated that a lot, that they were willing to do that”.

*Participant with employment*

### Theme 8: Proactivity Versus Passivity/Fatigue

The theme *Proactivity* clearly emerged from the groups with paid employment, and was absent in the groups without. Here, participants’ remarks showed many had a strong drive to be employed, and were active in persuing their goals. Often they wanted to grow professionally, which showed for instance from their efforts to actively seek feedback on their performance. Many indicated that they prepared job interviews well. When encountering problems at work, they often proactively found creative solutions to solve them.

“I had just recovered from a burnout episode [..] and I had to go find a new job. […] But I felt a huge barrier to apply for jobs […] and answer questions like ‘Why should we hire you’? [..] I really did not feel like those kind of questions and mind games. I started to have coffee with a lot of different people, to see if we connected. [..] I have a lot of ‘friends’ on LinkedIn..[..]. So I just went for a low threshold cup of coffee. Just coffee”.

*Participant with employment*

In contrast, in the groups without employment several participants indicated to feel disabled in their functioning because of a lack of energy. Fatigue and not being able to handle additional stress were reasons for them not to pursue finding paid employment.

### Theme 9: Communication

Mutually good communication between supervisor and autistic worker about the worker’s needs was a facilitator for sustainable employment. Communication challenges could also act as a barrier, particularly in two areas. First, participants in both groups often indicated to find job applications (especially interviews) difficult because of the *implicit social rules and expectations*. Many said they were not good at ‘selling themselves’ and therefore job interviews made them feel highly uncomfortable. Engaging in job interviews was experienced as ‘acting in a play’ which made them feel angry and exhausted. In addition, there were concerns about whether they would correctly understand and interpret the employers’ communication, and therefore the interviews often caused them to feel insecure.

“If someone asks a question with a certain intonation [..] you have to think first: ‘What does she mean by that?’ [..] I did not know she meant it as a joke.[..] it lowers your chances [of getting hired..] It kills me not to know how I came across, after a job interview.”

*Participant without employment*

“I have spent three years looking for work. Sevenhundred job applications, 30 assessments. Each time an allmost perfect score, and still rejected. I was eventually hired at [a high tech company], where I have worked for nine years now. Same company, same job, same job discription, and it all goes succesfully. So it does not have anything to do with my work skills. My job application skills [however], yeah they are a disaster”.

*Participant with employment*

An second difficulty concerning social and communication challenges hampering sustainable employment was *(too much) honesty*. In both groups particants said they knew they were too honest, which was not only difficult during job interviews, but also could be difficult in relationships at work.

“I immediately told our director the truth [in front of my coworkers], and my coworkers were happy with it […] but the director kept on looking at me, [and I thought] oh boy”

*Participant without employment*

### Theme 10: Disability Benefits Trap

In the group without employment, many said that receiving long-term disability benefits was a relief as it freed them from financial stress and obligations (e.g. applying for jobs). Moreover, many had experienced poor mental health because of negative previous work experiences. Receiving benefits protected them from financial stress and from having to try and get hurt again in a new workplace. However, many also commented on how they would like to work again one day, but felt ‘trapped’ because they did not want to risk loosing their disability benefits.

“[I would like to work but] it is a risk for [..] my financial situation. I receive long term benefits now, and the chance that I can get a fixed employment contract for the hours that I can work is is really small and hard to find. A new job is also a risk because I don’t know if I can function there. If I fail I [..] I risk […] loosing my right to receive any benefits”

*(participant without paid employment)*

## Discussion

In the present study, the aim was to study barriers and facilitators for sustainable employment in two separate groups of autistic adults: those *with* and *without* paid (competitive) employment. A total of ten themes and thirty-four subthemes were found, which illustrates that a large variety of factors are involved, and many are interconnected. For instance, participants with employment who were doing well often reported a combination of several facilitating factors, such as working in a work environment where they were treated respectfully, with a supportive supervisor who allowed them to do interesting work. The results should therefore be read integrally, rather than focusing on only one factor to explain differences between the groups. Also, interventions should be tailored to the individual and his/her context, and address as many of the barriers and facilitators as possible. Themes facilitating sustainable employment included the importance of a positive workplace atmosphere, a good supervisor, being able to do work that aligns with interests and talents, favorable working conditions, coaching, higher self-insight, higher self-esteem, and proactivity. Most themes and subthemes emerged from both groups, which highlights their importance, as the analyses were conducted separately for both groups. Differences between the groups were that those *with* paid employment seemed to have experienced more friendly workplaces and supervisors, received better coaching in finding and keeping employment, had higher self-insight and higher self-esteem, were more assertive and proactive.

Overall, when looking at all the themes and subthemes integrally, together the findings suggest there are two promising key areas of improvement for sustainable employability of autistic adults. The first key area is to improve their self-insight regarding what they need for positive wellbeing, and self-knowledge regarding talents and wishes. Also, better self-insight into stressors that negatively affect well-being, or regarding personal boundaries that help protect their well-being, will improve sustainable employment. Previous studies have found that emotional self-awareness can be lower in autistic adults [14], and that they may have difficulty to identify both their need for adjustments and the specific adjustments that might best support them [15]. Without knowing the causes of challenges, it is hard to find solutions to deal with them. In the groups with paid employment, many said that having learned practical solutions to previous problems contributed to their sustainable employment, also after coaching. Moreover, increased self-knowledge about personal boundaries may safeguard a healthy work-life balance and protect against burn-out. Many respondents reported having worked too hard, which had left them feeling depleted and burned-out. Furthermore, our findings also suggest that it is important to increase self-knowledge regarding needs for good wellbeing in an early stage in autistic peoples’ lives, to avoid negative experiences. For instance, participants without employment often had lower self-esteem and had higher distrust of supervisors and coworkers because of previous damaging social experiences. In addition, they often lacked the proactivity and motivation to search for a new job, which was a theme the groups differed on. Finally, many participants had truly suffered from the fact they had never understood why they were struggling so much in their lives, and for many of those in the groups without employment, finally getting the diagnosis was perceived as an acknowledgement for the challenges and pain they had experienced and a reason never to want to go back to the workforce again. Hence, our findings suggest that early self-insight into needs and stressors may increase well-being and prevent later mental ill health and unemployment. Future research should study the role of self-reflection skills in sustainable employability and how they can be enhanced, e.g. by supervisors or occupational professionals.

A final reason why it is important to increase self-insight is that it also facilitates finding a job that aligns with interest and skills. Having interesting work was seen as an important facilitator for sustainable employment. Autistic individuals may have difficulty recognizing their strengths in the workplace and may require support in identifying and communicating their strengths to others [16, 17]. Previous studies in non-autistic populations have also shown that for well-being and sustainable employment, it is important that workers can do work that they value and find interesting [18, 19]. Whereas a good person-job fit is important for successful employment outcomes [20], many participants without employment did not know what their interests or talents were, which makes it more difficult to find a job that matches well. Participants without paid employment often had received no (adequate) support, whereas good coaches can empower, can help to increase self-insight, can support in job crafting and improving the job-person fit, and can support in assertive communication. This indicates a clear area for improvement.

The second key area for improvement of sustainable employment rates of autistic adults is that workplaces should become more friendly, well-being oriented, and inclusive. Thirteen of the 34 subthemes were related to a good workplace atmosphere and positive supervisor attitudes. Previous studies have illustrated the important role of the work environment for sustainable employment of autistic adults, including workplace stakeholders’ attitudes, knowledge, understanding and support, especially of line managers, e.g. [16, 21–28]. Specifically, others have suggested that autism awareness and acceptance need to be improved, stigma and discrimination needs to be eradicated [26–28] and that work adjustments and support should be tailored to the *individual* needs of the autistic worker [15, 29, 30].

It should be noted that many of the subthemes found corresponded with well-known workplace preconditions for optimal wellbeing and sustainable employment in the general working population, e.g. autonomy [31], authenticity [32], use of talents, skills, and interests [33], job coaching [34] and jobcrafting [35]. A recent systematic review showed that the workplace psychosocial safety climate directly affects health, well-being, safety, and performance of workers [36]. Hence, employers who invest in these aspects and who create healthy and decent workplaces will not only benefit autistic adults but will enhance well-being and sustainable employment of all their workers.

This study has several limitations. First, two types of participants were recruited: those with paid employment, and those without. This design ignored other relevant subgroups e.g. based on employment history, or high or low well-being at work. So far, most literature has focused on problematic occupational outcomes, rather than on what autistic adults need to thrive and be happy at work. Even though some recent studies did have such a positive focus (e.g. [6, 21, 28]) more studies from this perspective are needed. Also, participants were only eligible if they had received an Autism Spectrum Disorder diagnosis from a psychologist or psychiatrist. This implies that they must have visited these professionals for mental health issues, and that autistic adults without a formal diagnosis who never sought help because they were doing well were not included. Moreover, the study population consisted of Caucasian participants only and was highly educated.

## Conclusions

In conclusion, a large variety of barriers and facilitators affect sustainable employment of autistic adults, and many are interconnected. Themes facilitating sustainable employment included the importance of a positive workplace atmosphere, a good supervisor, being able to do work that aligns with interests and talents, favorable working conditions, good coaching, higher self-insight, higher self-esteem, and proactivity. Overall, the results suggest that two key areas should both be addressed to improve sustainable employment rates of autistic adults: First, to realize more friendly, well-being oriented and inclusive workplaces, and second to increase autistic adults’ self-insight into personal needs for well-being and self-knowledge regarding talents, wishes and well-being boundaries.

## Supplementary Information

Below is the link to the electronic supplementary material.

Supplementary file1 (DOCX 18 kb)

## Data Availability

The datasets generated during the current study are available from the corresponding author on reasonable request.
